# Recent Studies on DNA Adducts Resulting from Human Exposure to Tobacco Smoke

**DOI:** 10.3390/toxics7010016

**Published:** 2019-03-19

**Authors:** Bin Ma, Irina Stepanov, Stephen S. Hecht

**Affiliations:** Masonic Cancer Center, University of Minnesota, Minneapolis, MN 55455, USA; stepa011@umn.edu (I.S.); hecht002@umn.edu (S.S.H.)

**Keywords:** DNA adducts, tobacco smoke, human carcinogen, biomarkers, cancer risk, mass spectrometry

## Abstract

DNA adducts are believed to play a central role in the induction of cancer in cigarette smokers and are proposed as being potential biomarkers of cancer risk. We have summarized research conducted since 2012 on DNA adduct formation in smokers. A variety of DNA adducts derived from various classes of carcinogens, including aromatic amines, polycyclic aromatic hydrocarbons, tobacco-specific nitrosamines, alkylating agents, aldehydes, volatile carcinogens, as well as oxidative damage have been reported. The results are discussed with particular attention to the analytical methods used in those studies. Mass spectrometry-based methods that have higher selectivity and specificity compared to ^32^P-postlabeling or immunochemical approaches are preferred. Multiple DNA adducts specific to tobacco constituents have also been characterized for the first time in vitro or detected in vivo since 2012, and descriptions of those adducts are included. We also discuss common issues related to measuring DNA adducts in humans, including the development and validation of analytical methods and prevention of artifact formation.

## 1. Introduction

Cigarette smoking causes multiple types of cancers. Despite the advent of advanced cancer genomics and impressive targeted therapies, there were still an estimated 9.6 million cancer deaths worldwide in 2018 with 22% of those deaths being caused by cigarette smoking [1]. In the U.S., where lung cancer is the leading cause of cancer death in both men and women, an estimated 154,000 lung cancer deaths occurred in 2018, with 90% being caused by cigarette smoking [2,3]. Not all smokers develop cancer. While 90% of all lung cancer-related deaths in the U.S. are attributable to cigarette smoking, only 24% of male smokers and 11% of female smokers may die from lung cancer over their lifetime, assuming that there is no competing cause of death [4,5]. A significant research challenge is to identify those smokers who have higher cancer risk, so prevention approaches can be initiated at an early stage before too much damage has been done.

One strategy to address this challenge is to identify and validate smoking-related biomarkers associated with tobacco exposure and cancer risk. We have demonstrated the association of certain urinary tobacco smoke biomarkers with lung cancer risk, independent of smoking intensity and duration. These biomarkers include cotinine and its glucuronide—metabolites of nicotine; 4-(methylnitrosamino)-1-(3-pyridyl)-1-butanol (NNAL) and its glucuronides—metabolites of a tobacco specific lung carcinogen 4-(methylnitrosamino)-1-(3-pyridyl)-1-butanone (NNK); and *r*-1-,t-2,3,c-4-tetrahydroxy-1,2,3,4-tetrahydrophenanthrene (PheT), a biomarker of polycyclic aromatic hydrocarbon (PAH) exposure and metabolic activation [6,7]. Similarly, levels of a urinary biomarker of exposure to another tobacco-specific carcinogen -*N*′-nitrosonornicotine (NNN)- have been independently and prospectively associated with the risk of esophageal cancer in smokers [8]. In addition, low activity forms of cytochrome P450 2A6, the major nicotine metabolizing enzyme, have been related to decreased lung cancer risk in cigarette smokers [9]. All of these parameters are related to carcinogen uptake by smokers, but do not provide information on the next critical step in the carcinogenic process—DNA adduct formation. DNA adducts associated with tobacco smoke exposure have the potential to significantly contribute to our understanding of the cancer process and could possibly be biomarkers of cancer risk.

**Figure 1:**
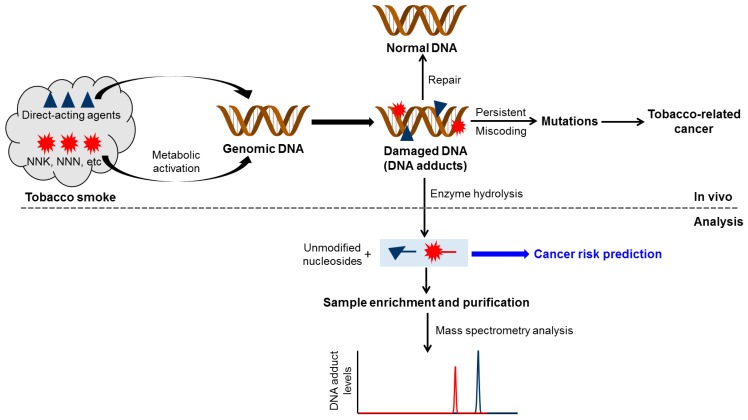
An overview of the central role of DNA adduct formation in tobacco-related cancer. DNA damage also leads to cell apoptosis. A more detailed mechanistic framework was illustrated by Hecht S.S. [13].

DNA adducts are compounds formed when chemicals react with DNA. Figure 1 provides an overview of the central role of DNA adduct formation in tobacco-related cancer. Tobacco smoke contains a highly complex mixture of over 7000 characterized chemical compounds; certain chemicals such as formaldehyde and acetaldehyde are reactive and directly bind to DNA, while some others, including NNK, NNN, and PAH require metabolic activation to reactive intermediates capable of reacting with DNA. The resulting covalent DNA addition products are commonly referred to as “DNA adducts” (Figure 1). Because of the DNA repair enzymes present in the body, the DNA adducts can be removed and the DNA returned to normal. However, if the repair process is overwhelmed or not completely efficient, the DNA adducts can persist and potentially cause miscoding during DNA replication. If the miscoding events occur in critical genes such as *p53* and *RAS*, they can result in the loss of normal growth control mechanisms, and eventually, the development of cancer. Therefore, DNA adduct formation plays a critical role in tobacco smoke-related cancer development. The general protocol to measure DNA adducts includes isolating DNA from biological samples, hydrolyzing the DNA using a cocktail of enzymes, purifying the hydrolyzed samples and enriching the DNA adducts, and then analyzing them by various techniques such as mass spectrometry. The ultimate goal is to quantify these tobacco smoke-related DNA adducts in human biological samples and to incorporate the results into epidemiologic studies to evaluate and validate their role in cancer risk prediction (Figure 1). While this challenging goal has yet to be reached, constantly improving methods for DNA adduct quantitation, as discussed here, provide at least a potential path. We also note that reactive tobacco chemicals can modify other macromolecules such as protein, which can also result in significant biological consequences and can be used in bio-monitoring [10,11].

In 2002, Phillips reviewed previous studies on smoking-related DNA adduct formation in human tissues [12]. A large number of studies mentioned in that review used ^32^P-postlabeling or immunochemical approaches for the analysis of DNA adducts. In many cases, “bulky DNA adducts” were reported but it was not clear which specific DNA adducts were being measured. Nevertheless, the effects of smoking were still evident by the detection of elevated levels of DNA adducts in many human tissues [12]. In 2012, Phillips updated this topic and reviewed studies on tobacco smoke-related DNA adducts published since 2002 [10]. That review covered the chemical nature and origins of smoking-related DNA adducts and the effect of DNA repair and gene polymorphisms on the levels of DNA adducts in humans. Many studies cited in that review demonstrated convincing evidence of higher levels of certain adducts in smokers than nonsmokers. In the same published issue as Phillips’ paper, we also reviewed and discussed structurally characterized DNA adducts in the lungs of smokers and their potential role in lung carcinogenesis by tobacco smoke [13].

**Figure 2:**
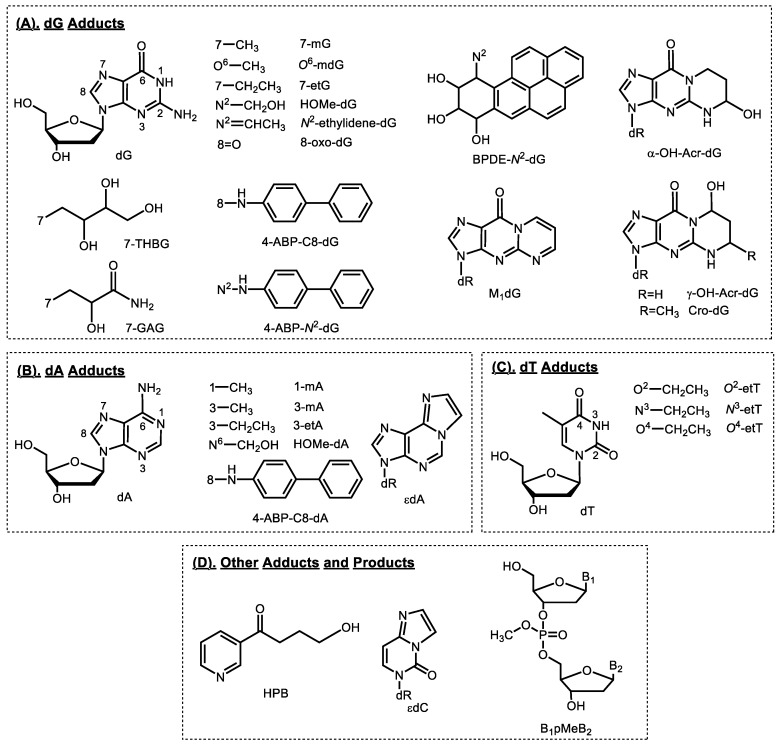
Structures of DNA adducts detected in humans. dR, 2′-deoxyribose; HPB, 4-hydroxy-1-(3-pyridyl)-1-butanone.

**Figure 3:**
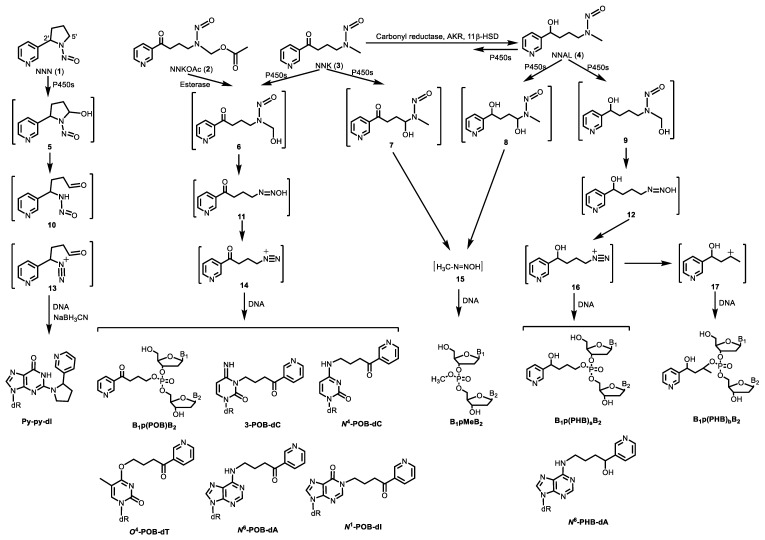
NNK and NNN-derived DNA base and phosphate adducts. NNK and NNN form various DNA adducts, and only newly characterized adducts or adducts detected for the first time in vivo since 2012 are presented. NNN is also metabolized via 2’-hydroxylation to intermediate **14**, which reacts with DNA to form POB base adducts. B_1_ and B_2_ represent the same or different nucleobases. NNK, 4-(methylnitrosamino)-1-(3-pyridyl)-1-butanone; NNN, *N*′-nitrosonornicotine; POB, pyridyloxobutyl; dR, 2′-deoxyribose.

This review covers 1) studies on formation of tobacco smoke-related DNA adducts in humans (Figure 2) since Phillips’ review in 2012; and 2) newly characterized DNA adducts formed by tobacco-specific chemicals (i.e., NNK and NNN) in in vitro and in vivo models (Figure 3). Future directions of applying DNA adducts as biomarkers of tobacco smoke exposure and associated cancer risk are also discussed.

## 2. Smoking-Related DNA Adducts in Humans

### 2.1. Tobacco-specific Nitrosamines (TSNA)

The most studied TSNA are NNK and NNN, which are considered “carcinogenic to humans” by the International Agency for Research on Cancer [14]. NNK causes lung tumors in all species tested, independent of the route of administration [15]. NNN induces tumors of the oral cavity, esophagus, and nasal cavities in rats and respiratory tract tumors in mice, mink, and Syrian golden hamsters [15]. NNK and NNN are present in all tobacco products. Both compounds are specific to tobacco and are not found in any other product, except that NNN can also be formed endogenously from nornicotine, a minor tobacco alkaloid and a nicotine metabolite [16,17,18].

NNK and NNN are metabolically activated to intermediate **14** (Figure 3), which reacts with DNA to form pyridyloxobutyl (POB) adducts at the four nucleobases and the oxygens of the phosphate backbone (Figure 3) [7,19,20,21,22]. Under acid or neutral thermal hydrolysis, most of the POB base adducts release 4-hydroxy-1-(3-pyridyl)-1-butanone (HPB, Figure 2). Multiple in vitro and animal studies demonstrated the relationship of HPB-releasing DNA adducts to NNK and NNN dose as well as biomarkers of NNK exposure [15]. HPB-releasing DNA adducts were also detected by our group and others in human tissues including lung, tracheobronchus, esophagus, and cardia [23,24,25]. We demonstrated for the first time the presence of HPB-releasing DNA adducts in human lung, with adduct levels being higher in smokers than nonsmokers [25]. Similar results were observed by Schlobe, et al, with HPB levels in the lung being significantly higher in 21 self-reported smokers compared to that in 11 self-reported nonsmokers [24]. However, another study by the same group showed no difference of adduct levels between smokers and nonsmokers [23]. All these studies had a relatively small sample size, probably due to the limited availability of human tissues, which had to be obtained by surgery. Obtaining these human tissues from individuals for smoking exposure evaluation and HPB-releasing DNA adduct analysis is highly impractical in larger studies. Surrogate tissues, which can be less invasively obtained, should be explored in such studies.

Exfoliated oral mucosa cells are relatively simple and noninvasive to be collected, and could be an excellent source of surrogate tissue for evaluating smoking exposure and adduct formation [26,27]. We developed a robust and sensitive liquid chromatography (LC)−electrospray ionization (ESI)−tandem mass spectrometry (MS/MS) method for the analysis of HPB-releasing DNA adducts in human oral cells (Table 1) [28]. Oral cells were collected from 30 smokers and 15 nonsmokers using a mouthwash rinse or buccal brushing with a cytobrush. In the oral cells collected by mouthwash, HPB was detected in 20 out of 28 smoker samples with quantifiable DNA yield, and in 3 out of 15 nonsmoker samples. Higher levels of HPB-releasing DNA adducts were observed in smokers with an average of 12 pmol adducts/mg DNA, compared to nonsmokers with an average of 0.23 pmol adducts/mg DNA. In the buccal brushing samples collected from the same 30 smokers, HPB was detected in 24 out of 27 samples with quantifiable DNA yield, averaging 45 pmol adducts/mg DNA. A correlation of adduct levels (*R* = 0.73, *p* < 0.0001) was also observed between buccal burshings and mouthwash samples from smokers [28]. The LC−ESI−MS/MS method we developed in that study demonstrated the applicability to the analysis of oral cell samples collected by mouthwash or buccal brushing. However, application of this method in studies in which only limited oral cells are available for DNA extraction required further optimization to increase its sensitivity and selectivity.

We then modified the method by optimizing the sample preparation procedure, and more notably by switching from LC−ESI−MS/MS to LC-nanoelectrospray ionization (NSI)−high-resolution tandem mass spectrometry (HRMS/MS) [29]. The LC-NSI-HRMS/MS-based methods for DNA adduct analysis have been very successful in our studies of other DNA adducts [20,30,31,32,33,34,35,36]. Using the optimized method, the sensitivity was improved seven-fold compared to the previous LC−ESI−MS/MS method, achieving a limit of quantitation (LOQ) of 0.35 fmol on-column. More importantly, the baseline noise commonly observed in mass spectrometry without high resolution capability was completely eliminated, greatly improving the selectivity of the method. We then applied this method to the analysis of HPB-releasing DNA adducts in oral cells collected by buccal brushing from 65 smokers, including 30 head and neck squamous cell carcinoma (HNSCC) patients and 35 cancer-free controls. HPB-releasing DNA adducts were detected in 29 out of 30 samples of HNSCC patients averaging 8.2 pmol adducts/mg DNA, compared to 20 out of 35 samples of cancer-free smokers averaging 4.5 pmol adducts/mg DNA. The median HPB-releasing DNA adduct level was 6.6 times greater for smokers with HNSCC than for those without HNSCC (*p* = 0.002) [29]. These results suggest that HPB-releasing DNA adducts may play a critical role in the development of smoking-induced HNSCC, and can potentially be used to identify susceptible smokers.

### 2.2. Bulky/Aromatic Adducts

The terminology “bulky DNA adducts” comes from early studies using a ^32^P-postlabeling approach to measure DNA adducts formed by high molecular weight chemical carcinogens, including PAH and probably some other aromatic and nonpolar chemicals [37,38]. PAH are formed primarily by incomplete combustion of tobacco and other organic components during smoking. PAH are also released from burning coal, oil, gasoline, wood, and are present in air, soil and water. Studies on bulky DNA adducts, sometimes referred to as PAH-DNA adducts, were comprehensively covered by Phillips et al. in 2002 [12] and 2012 [10]. The overall trend was that higher adduct levels were observed in smokers compared to nonsmokers. Since 2012, continuous efforts have been made to measure bulky DNA adducts in human samples including lung and leukocytes (Table 1) [39,40,41,42,43,44,45,46,47,48,49,50,51,52]. Smoking is a contributor to adduct formation in these studies. However, since the ^32^P-postlabeling technique was exclusively used as the detection method, structural information for these adducts was not available, which prevents one from working backwards to evaluate the exposure to responsible chemicals.

One of the PAH adducts is BPDE-*N*^2^-dG (Figure 2), which is formed by BPDE, a metabolite of the most studied carcinogenic PAH—benzo[*a*]pyrene (BaP). In humans, BPDE-*N*^2^-dG was reported to be present in buccal cells [53], sputum [53], lung [53,54], leukocytes [55], lymphocytes [56], tonsil [57], oropharynx [57], gingiva [57], and prostate [58] using immunochemical methods. Another method to measure this adduct is to hydrolyze the DNA in acid and analyze BaP-7,8,9,10-tetraols instead of the intact adducts by LC-fluorescence or gas chromatography/mass spectrometry methods [59,60]. However, the specificity of these methods, although in some cases well designed and validated, has to be considered when interpreting the data. This concern was further highlighted by studies using mass spectrometry-based methods [61,62,63,64]. In one study analyzing human lung tissues, BPDE-*N*^2^-dG was not detected in any of the 10 samples with a limit of detection (LOD) of one−three adducts per 10^8^ nucleotides [61], and the second study showed detectable adducts in only 1 out 26 human lung DNA samples (LOD, 0.3 adducts per 10^8^ nucleotides) [62]. BPDE-*N*^2^-dG was not detected in salivary or oral cell DNA in another two studies [63,64]. Finally, a recent study failed to detect this adduct in human prostate tissue [65].

We recently developed an ultrasensitive LC-NSI-HRMS/MS method for the analysis of BPDE-*N*^2^-dG in human lung DNA, with an LOD of 1 adduct per 10^11^ nucleotides, which is equivalent to 1 adduct per 10 human lung cells (Table 1) [32]. To our knowledge, this is the most sensitive DNA adduct quantitation method yet reported. We applied this method to the analysis of lung samples obtained during surgery for lung cancer from 29 patients. Another unique feature of this study was confirmation of smoking status at the time of lung cancer surgery by the measurement of urinary cotinine and NNAL. BPDE-*N*^2^-dG was detected in 20 out of 29 samples, with smoker and nonsmoker DNA containing 3.1 and 1.3 adducts per 10^11^ nucleotides, respectively. The adduct levels in our study are in contrast to those obtained by the immunochemical approach, with levels typically ranging from 1–30 adducts per 10^8^ nucleotides in lung tissues [53,54]. Compared to mass spectrometry-based methods including our study, significantly higher levels (~1,000-fold) of adducts were observed by immunochemistry-based methods. Although the lung samples analyzed in these studies were from different subjects, the significantly high level observed by immunochemistry-based methods suggests that those methods should be re-evaluated to avoid false-positive results, and the data obtained from those studies should be interpreted with caution. Studies should be conducted using a mass spectrometry-based method and an immunochemistry-based method to analyze the same human lung samples, to compare the levels of BPDE-*N*^2^-dG adducts assessed by the two methods.

### 2.3. Aromatic and Heterocyclic Aromatic Amines

Aromatic and heterocyclic aromatic amines are structurally related classes of chemicals that are present in tobacco smoke, formed in cooked meats, and occur as contaminants in the atmosphere [38,66]. One such compound, an aromatic amine 4-aminobiphenyl (4-ABP) is a known bladder carcinogen in both animals and humans [66]. The major DNA adduct formed by 4-ABP is 4-ABP-C8-dG, and to a lesser extent, 4-ABP-C8-dA and 4-ABP-*N*^2^-dG (Figure 2) [67].

In a study investigating the mutagenicity of 4-ABP-derived DNA adducts, the three adducts were measured in both normal human urothelial mucosa and bladder tumor tissues by ^32^P-postlabeling-based methods (Table 1) [68]. All three adducts were detected in the urothelial mucosa samples (*n* = 19) with levels ranging from 2.3–12, 4.6–16, and 2.3–12 adducts per 10^8^ nucleotides for 4-ABP-C8-dG, 4-ABP-C8-dA, and 4-ABP-*N*^2^-dG, respectively. The three adducts were also detected in bladder tumor samples (*n* = 10) with levels ranging from 2.3–9.2, 4.6–25, and 2.3–28 adducts per 10^8^ nucleotides for 4-ABP-C8-dG, 4-ABP-C8-dA, and 4-ABP-*N*^2^-dG, respectively. No significant difference was observed in either total adduct levels or individual adduct levels between normal human urothelial mucosa and bladder tumor tissue [68].

In contrast to the results obtained by ^32^P-postlabeling methods, studies using mass spectrometry-based methods showed much lower levels or no detection of 4-ABP adducts in human samples [51,65,67]. Xiao, et al developed and validated an LC-NSI-HRMS/MS method for the analysis of 4-ABP-C8-dG with an LOQ of 0.22 adducts per 10^8^ nucleotides using 2.5 µg DNA. The method was applied to the analysis of normal tumor-adjacent prostate tissues from 35 prostate cancer patients. 4-ABP-C8-dG was detected at a level of 2.8 adducts per 10^8^ nucleotides in only one out of 35 samples and that one patient was identified as a nonsmoker [65]. The same research group subsequently applied this method to the analysis of normal tumor-adjacent bladder mucosa tissues from bladder cancer patients. 4-ABP-C8-dG was detected in 12 out of 41 samples with levels ranging from 0.14 to 3.4 adducts per 10^8^ nucleotides. 4-ABP-C8-dA and 4-ABP-*N*^2^-dG were also detected in the same samples of two subjects who had the highest levels of 4-ABP-C8-dG [67]. In another study, Gu, et al applied an LC-MS/MS^3^ method to the analysis of tumor-adjacent normal mammary tissues from 70 breast cancer patients. No 4-ABP-C8-dG was detected in any of the samples with an LOQ of 3 adducts per 10^8^ nucleotides [51]. In comparison, an earlier study reported detecting 4-ABP adducts in all the 55 breast tissues by an immunochemical method [69], even though its sensitivity was ~100-fold lower compared to the LC-MS/MS^3^ method.

### 2.4. Methylating Agents

There are several methylating agents present in tobacco products, including the tobacco-specific nitrosamine NNK, *N*-nitrosodimethylamine (NDMA) and methyl chloride. All of them can lead to formation of methyl DNA adducts. NNK and its metabolite NNAL are metabolized to methane diazohydroxide **15** and then methyldiazonium ion (Figure 3), which reacts with DNA forming methyl DNA base adducts including 7-methylguanine (7-mG), *O*^6^-methyldeoxyguanosine (*O*^6^-mdG) (Figure 2) [15] and methyl DNA phosphate adducts [34]. The major mutagenic and toxic methyl base adduct is *O*^6^-mdG, which induces primarily GC→AT transition mutations, with some other minor methyl base adducts also demonstrating mutagenicity and cytotoxicity [70]. In addition to methyl base adducts, some methylating agents also react with the DNA phosphate backbone to form methyl DNA phosphate adducts—B_1_pMeB_2_ (Figure 2, see details in Section 3.1). B_1_pMeB_2_ adducts were demonstrated to induce TT→GT and TT→GC mutations [71], inhibit RNA synthesis [72], and affect the binding affinity of DNA to other macromolecules [73].

Methyl DNA base adducts were detected in human lung tissue samples [53,74]. *O*^6^-mdG was measured in the tumor-adjacent normal lung tissue samples from lung cancer patients by an immunochemical approach, and no difference in adduct levels was observed between smokers (*n* = 41) and nonsmokers (*n* = 13) (Table 1) [53]. In another study, 7-mG was detected by an immunochemical approach in lung samples from 14 former and 6 current smokers undergoing surgery for lung cancer [74]. The lung tissues were collected from five different positions of the lung including central bronchus, lung periphery, and three equidistant points along its length. The levels of 7-mG in all the samples averaged 0.75 ± 0.57 adducts per 10^6^ dG. No significant difference in adduct levels was observed at different lung positions. Compared to former smokers, the levels of 7-mG were higher (*p* = 0.047) in current smokers at two lung positions including the lung periphery [74].

Multiple methyl DNA base adducts were also detected in human urine samples [75,76,77,78]. Wang, et al developed a capillary LC-HRMS/MS method for the simultaneous analysis of 7-mG, 3-methyladenine (3-mA), and 1-methyladenine (1-mA) in human urine samples (Figure 2 and Table 1). They applied the method to the analysis of urine samples from 20 smokers and 14 nonsmokers. The levels of the three adducts were all significantly higher in smokers than nonsmokers, with the difference in 3-mA levels being the most significant (11-fold, *p* < 0.0001) [75]. Higher levels of 3-mA in smokers compared to nonsmokers were also observed in another two studies that used LC-MS/MS-based analytical methods [76,77]. In one study, the level of 3-mA was also correlated with the level of urinary NNAL in smokers (*n* = 192, *r* = 0.48, *p* < 0.001) [77]. The correlation was also observed between adduct (3-mA and 7-mG) levels and urinary NNAL in another study [78]. However, the levels of these methyl adducts detected in urine are far greater than the levels of adducts possibly formed by NNK, suggesting a contribution of methylating agents from other sources such as diet.

We have developed an ultrasensitive LC-NSI-HRMS/MS method for the analysis of methyl DNA phosphate adducts (B_1_pMeB_2_, Table 1 and Figure 2) in human lung DNA [79]. The adduct levels were measured in both tumor and adjacent normal tissues from 30 lung cancer patients, including 13 current smokers and 17 current nonsmokers, as confirmed by measurements of urinary cotinine and NNAL. Levels of total B_1_pMeB_2_ in normal lung tissues were higher (*p* < 0.05) in smokers than nonsmokers, with an average of 13 and 8 adducts per 10^9^ nucleotides, respectively. More details on DNA phosphate adducts, including B_1_pMeB_2_, are discussed in Section 3.1.

### 2.5. Ethylating Agents

Studies have demonstrated the presence of direct-acting ethylating agent(s) in tobacco products and tobacco smoke, though without characterized structure(s). These agents can react with DNA forming ethyl DNA base adducts [80,81]. Compared to methyl DNA base adducts, ethyl DNA base adducts are generally more persistent in vivo with more specificity to smoking [82,83,84]. Certain ethyl DNA base adducts, for example *O*^4^-ethylthymidine (*O*^4^-etT) (Figure 2), are promutagenic lesions and may contribute to the initiation of hepatocellular carcinomas in animal models [85]. Ethyl DNA base adducts were reported to be present in human leukocytes [84,86], saliva [83,87], and urine samples [76,77,88].

Chen et al. developed a series of LC-NSI-MS/MS methods for the analysis of multiple ethyl DNA base adducts in human biological samples (Table 1) [83,84,86,87,88]. Two of their studies measured these DNA adducts in human leukocytes. In one study, three ethyl DNA base adducts, *O*^2^-ethylthymidine (*O*^2^-etT, Figure 2), *N*^3^-ethylthymidine (*N*^3^-etT), and *O*^4^-etT, were detected and quantified in leukocyte DNA samples from 20 smokers and 20 nonsmokers [86]. The levels of *O*^2^-etT, *N*^3^-etT and *O*^4^-etT in smokers were 45 ± 52, 41 ± 44, and 48 ± 54 adducts per 10^8^ nucleotides, while nonsmokers had significantly lower (*p* < 0.001) adduct levels at 0.19 ± 0.87, 4.1 ± 13, and 1.0 ± 2.9 adducts per 10^8^ nucleotides, respectively. Furthermore, the level of *O*^2^-etT was correlated with the smoking index (number of cigarettes per day × years smoked) (*r* = 0.48, *p* < 0.05) [86]. In the other study, 3-ethyladenine (3-etA), and 7-ethylguanine (7-etG) were detected and quantified in leukocyte DNA samples from 20 smokers and 20 nonsmokers. The levels of 3-etA and 7-etG in smokers were 16 ± 7.8 and 9.7 ± 8.3 adducts per 10^8^ nucleotides, significantly higher (*p* < 0.0001) than the levels in nonsmokers at 5.4 ± 2.6 and 0.3 ± 0.8 adducts per 10^8^ nucleotides, respectively. Additionally, the levels of both adducts were correlated with the smoking index [84]. Chen et al. also measured ethyl DNA base adducts in human saliva. In one study, *O*^2^-etT, *N*^3^-etT and *O*^4^-etT were detected in saliva samples from 20 smokers at 5.3 ± 6.2, 4.5 ± 5.7, and 4.2 ± 8.0 adducts per 10^8^ nucleotides, respectively, while none of the adducts were detected in saliva samples from 13 nonsmokers [83]. In another study, 3-etA and 7-etG were detected and quantified in saliva samples from 15 smokers and 15 nonsmokers. The levels of 3-etA and 7-etG in smokers were 13 ± 7.0 and 14 ± 8.2 adducts per 10^8^ nucleotides, significantly higher (*p* < 0.0001) than in nonsmokers with adduct levels at 9.7 ± 5.3 and 3.8 ± 2.8 adducts per 10^8^ nucleotides, respectively. In addition, the levels of 7-etG in smokers were correlated with the number of cigarettes per day (*r* = 0.76, *p* < 0.0001) and the smoking index (*r* = 0.85, *p* < 0.0001) [87].

Ethyl DNA base adducts have also been reported to be present in human urine samples (Table 1) [76,77,88]. In addition to leukocytes and saliva, Chen, et al also developed an LC-NSI-MS/MS method for the analysis of 3-etA and 7-etG in urine samples from 21 smokers and 20 nonsmokers. 3-etA and 7-etG were detected in all smokers with levels at 69 ± 29 and 19 ± 14 pg/mL urine, respectively. In nonsmokers, the adducts were detected in 16 out of 20 samples, with levels at 3.5 ± 3.8 and 2.4 ± 3.0 pg/mL urine for 3-etA and 7-etG, respectively. Higher adduct levels were observed in smokers compared to nonsmokers [88]. Similar results were also observed in another two studies that measured 3-etA and 7-etG in urine samples [76,77].

### 2.6. 1,3-Butadiene

1,3-Butadiene is a colorless gas that is widely used in the polymer industry and is present in cigarette smoke, the urban environment, and automobile exhaust. 1,3-Butadiene is metabolized to epoxides, which react with DNA forming DNA adducts with the most abundant one in animals being 7-(2, 3, 4-trihydroxybut-1-yl) guanine (7-THBG, Figure 2) [89]. Sangaraju et al. developed a capillary LC-HRMS/MS method for the analysis of 7-THBG in human leukocytes (Table 1) [90]. The method was first applied to the analysis of leukocyte samples from 13 smokers and 13 nonsmokers. The levels of 7-THBG were 0.82 ± 0.51 and 0.71 ± 0.53 adducts per 10^8^ nucleotides in smokers and nonsmokers, respectively, a non-significant difference (*p* = 0.6). To further evaluate the influence of smoking on adduct formation, the adduct levels were determined in 10 individuals who participated in a smoking cessation study. The leukocyte samples were collected before smoking cessation, and 28 and 84 days afterwards. The adduct level was not significantly changed by cessation. In the same study, the influence of occupational exposure to 1,3-butadiene on adduct levels was also investigated. 7-THBG was measured in leukocyte samples from 10 workers with known exposure levels and 10 matched controls. Compared to the controls (0.31 ± 0.22 adducts per 10^8^ nucleotides), the levels of 7-THBG were significantly elevated in the exposed workers (0.97 ± 0.38 adducts per 10^8^ nucleotides, *p* < 0.001) [90].

### 2.7. Acrolein

Human exposure to the α,β-unsaturated acrolein can be from tobacco smoke, automobile exhaust, plastic waste, heated cooking oil and endogenous lipid peroxidation [91]. Acrolein reacts with deoxyguanosine in DNA to form two pairs of regioisomeric 1,*N*^2^-propanodeoxyguanosine adducts: (6*R*/*S*)-3-(2’-deoxyribos-1’-yl)-5,6,7,8-tetrahydro-6-hydroxypyrimido[1,2-*a*]purine-10(3*H*)one (α-OH-Acr-dG, Figure 2) and (8*R*/*S*)-3-(2’-deoxyribos-1’-yl)-5,6,7,8-tetrahydro-8-hydroxypyrimido[1,2-*a*]purine-10(3*H*)one (γ-OH-Acr-dG). Compared to γ-OH-Acr-dG, α-OH-Acr-dG is more mutagenic and predominantly induces G→T transversions.

We developed an LC-ESI-MS/MS method to analyze Acr-dG adducts in human leukocyte DNA (Table 1) and applied this method to the analysis of leukocyte DNA samples from 25 smokers and 25 nonsmokers [92]. γ-OH-Acr-dG was the predominant isomer in all samples, while α-OH-Acr-dG was detected in only one nonsmoker and two smokers. The total levels of α-OH-Acr-dG and γ-OH-Acr-dG were not different between smokers and nonsmokers, with levels averaging 0.74 ± 0.34 and 0.98 ± 0.55 adducts per 10^8^ nucleotides, respectively [92]. In an earlier study, we also measured Acr-dG adducts in human lung DNA from smokers and nonsmokers using an LC-ESI-MS/MS method [93]. However, the smoking status of some subjects in that study was unknown, which made it difficult to interpret the role of smoking in the adduct formation. Recently, we have developed a new ultrasensitive LC-NSI-HRMS/MS method for the analysis of Acr-dG adducts in human lung DNA samples [94]. In addition to improved sensitivity, we also managed to lower the levels of artifactual formation of adducts during sample preparation and minimized the interference of artifactually formed DNA adducts in quantitation. We analyzed the levels of Acr-dG adducts in lung DNA of 19 smokers and 18 nonsmokers who underwent surgery for lung cancer. The smoking status of these subjects was confirmed by urinary total cotinine and NNAL. The levels of α-OH-Acr-dG averaged 0.86 ± 0.27 and 1.0 ± 0.47 adducts per 10^8^ nucleotides in smokers and nonsmokers, respectively, and the levels of γ-OH-Acr-dG averaged 2.0 ± 1.4 and 1.5 ± 0.64 adducts per 10^8^ nucleotides in smokers and nonsmokers, respectively. There was no significant difference in the levels of α-OH-Acr-dG or γ-OH-Acr-dG between smokers and nonsmokers [94]. While the sample size was relatively small, our results indicate that acrolein is not a major etiological agent for cigarette smoking related DNA damage.

Li et al. developed an LC-ESI-MS/MS-based method for the analysis of Acr-dG adducts in human saliva samples (Table 1). γ-OH-Acr-dG was detected in all samples, and no difference of adduct levels was observed between smokers (*n* = 16) and nonsmokers (*n* = 16). α-OH-Acr-dG was not detected in any of the samples [95]. γ-OH-Acr-dG as the major acrolein-derived DNA adduct was also confirmed in human lung and liver tissues in another LC-ESI-MS/MS-based study [96]. Using an immunochemical approach, Weng et al. measured γ-OH-Acr-dG in multiple human tissues including buccal cells, sputum, and lung tissue samples [53]. The levels of γ-OH-Acr-dG in buccal cells from smokers (*n* = 33) were significantly higher (*p* < 0.0001) compared to nonsmokers (*n* = 17). Similarly, the adduct levels in sputum were higher levels (*p* < 0.05) in smokers (*n* = 22) compared to nonsmokers (*n* = 8). In the same study, γ-OH-Acr-dG was also detected in noncancerous lung tissues obtained from lung cancer patients with higher levels in smokers (*n* = 41) compared to nonsmokers (*n* = 13) [53]. Compared to our study of γ-OH-Acr-dG in human lung tissues [94], the levels obtained by Weng et al. were 10–20 times higher, suggesting possible artifactual formation of γ-OH-Acr-dG, which was not investigated in their study. Similarly, a drastic difference of BPDE-dG levels was also observed between our study and the study by Weng et al. The average level of BPDE-dG in smokers’ lung in our study was ~3 adducts per 10^11^ nucleotides [32], while the average level in their study was ~1000 times higher [53]. Due to the potential significant artifactual formation of adducts in that study, its results and conclusions are questionable. Another study employed both ^32^P-postlabelling and immunochemical methods for the analysis of α-OH-Acr-dG and γ-OH-Acr-dG in normal human urothelial mucosa and bladder tumor tissues. γ-OH-Acr-dG was the predominant isomer in both tissues, with higher levels observed in tumor tissue compared to normal tissue [68].

### 2.8. Formaldehyde

Formaldehyde is considered a human carcinogen and is widely present in the environment. It is mainly used in the production of industrial resins. It is also produced during cooking and cigarette smoking as well as by endogenous processes. Formaldehyde reacts with DNA and forms several DNA adducts and cross-links, with the most abundant one being *N*^6^-hydroxymethyldeoxyadenosine (HOMe-dA, Figure 2). We were the first group that demonstrated the presence of this specific formaldehyde-DNA adduct in humans [97].

Li et al. developed an LC-ESI-MS/MS-based method for the simultaneous analysis of two formaldehyde-derived DNA adducts, HOMe-dA and *N*^2^-hydroxymethyldeoxyguanosine (HOMe-dG, Figure 2), in human saliva samples (Table 1). HOMe-dA and HOMe-dG were measured after sodium cyanoborohydride (NaBH_3_CN) reduction as *N*^6^-methyldeoxyadenosine and *N*^2^-methyldeoxyguanosine, respectively. The levels of HOMe-dA were 996 ± 757 and 670 ± 524 adducts per 10^8^ nucleotides in smokers (*n* = 16) and nonsmokers (*n* = 16), respectively, while HOMe-dG was detected in a much lower level in the same samples, with 26 ± 21 and 20 ± 11 adducts per 10^8^ nucleotides being present in smokers and nonsmokers, respectively. No statistical difference of adduct levels was observed between smokers and nonsmokers [95].

### 2.9. Acetaldehyde and Crotonaldehyde

Acetaldehyde associated with the consumption of alcoholic beverages is carcinogenic to humans [91]. Acetaldehyde is ubiquitous in the human environment, and is one of the most prevalent carcinogens in cigarette smoke. It also occurs widely in fruits, vegetables, and cooked meat. Crotonaldehyde is also ubiquitously present in the environment. It is present in mobile source emissions, tobacco smoke, and other thermal degradation mixtures. Crotonaldehyde is mutagenic and carcinogenic. Both acetaldehyde and crotonaldehyde react with DNA forming a pair of diastereomeric adducts, (6*S*,8*S*)- and (6*R*,8*R*)-3-(2′-deoxyribos-1′-yl)-5,6,7,8-tetrahydro-8-hydroxy-6-methylpyrimido[1,2-*a*]purine-10(3*H*)one (Cro-dG, Figure 2). In addition to Cro-dG, acetaldehyde also reacts with DNA to generate its major DNA adduct, *N*^2^-ethylidene-deoxyguanosine (*N*^2^-ethylidene-dG). We have previously developed LC-ESI-MS/MS-based methods and detected both Cro-dG and *N*^2^-ethylidene-dG in human DNA [98,99].

Using an immunochemical approach, Weng et al. detected Cro-dG in human buccal cells, sputum, and lung tissue samples (Table 1) [53]. The levels of Cro-dG in buccal cells from smokers (*n* = 33) were significantly higher (*p* < 0.0001) compared to nonsmokers (*n* = 17). Similarly, the adduct levels in sputum were also higher (*p* < 0.05) in smokers (*n* = 22) compared to nonsmokers (*n* = 8). Cro-dG was also detected in noncancerous lung tissues obtained from lung cancer patients with higher levels in smokers (*n* = 41) compared to nonsmokers (*n* = 13) [53]. However, the levels of Cro-dG reached ~1,000 adducts per 10^8^ nucleotides in buccal cells and sputum. Such high levels are surprising, and the specificity of the method as well as artifactual formation during sample preparation should be evaluated. In a second study using an LC-ESI-MS/MS method, Cro-dG was detected in saliva samples from 16 smokers at 2.6 ± 2.1 adducts per 10^8^ nucleotides, while no Cro-dG was detected in any of the samples from nonsmokers [94]. In a third study, Cro-dG was analyzed in urine samples using an LC-ESI-MS/MS method to investigate exposure to urban air pollution. Higher adduct levels were observed in subjects (*n* = 47) exposed to air pollution with a median value of 21 fmol of Cro-dG per mg creatinine, compared to controls (*n* = 35) with a median value of 8 fmol of Cro-dG per mg creatinine (*p* < 0.05) [100].

The acetaldehyde-derived *N*^2^-ethylidene-dG is generally measured after reduction (e.g., by NaBH_3_CN) as *N*^2^-ethyl-dG (Table 1) [99]. In the second study mentioned above, *N*^2^-ethylidene-dG (as *N*^2^-ethyl-dG) was also measured in the same saliva samples, with levels of 6.2 ± 3.5 and 0.59 ± 0.89 adducts per 10^8^ nucleotides being present in smokers and nonsmokers, respectively. The difference between smokers and nonsmokers was not statistically significant [94]. The formation of *N*^2^-ethylidene-dG was also investigated in alcohol consumption-related studies [101,102,103].

### 2.10. Acrylamide

Acrylamide is a probable human carcinogen and is present in tobacco smoke and in carbohydrate-rich foods processed at high temperatures. Acrylamide is metabolized to glycidamide, which reacts with DNA to form a major DNA adduct, 7-(2-carbamoyl-2-hydroxyethyl) guanine (7-GAG, Figure 2). Huang, et al developed an LC-ESI-MS/MS method for the analysis of 7-GAG in human urine (Table 1). The method was applied to the analysis of urine samples from 30 smokers and 33 nonsmokers. The levels of 7-GAG ranged from 0.61–6.22 (mean: 1.4) and 0.36–3.0 (mean: 0.93) µg/g creatinine in smokers and nonsmokers, respectively, a non-significant difference. However, the urinary *N*-acetyl-S-(propionamide)-cysteine, a metabolite of acrylamide, showed significantly higher levels (*p* < 0.001) in smokers compared to nonsmokers [104]. The same research group investigated in another study the effect of occupational exposure to acrylamide on the formation of 7-GAG. The adduct was measured in urine samples from eight workers who were exposed to acrylamide and 36 controls. Higher levels (*p* < 0.001) of 7-GAG were observed in exposed workers, with adduct levels ranging from 1.0–13 (mean: 2.5) µg/g creatinine, compared to controls with adduct levels ranging from 0.20–0.93 (mean: 0.36) µg/g creatinine [105].

### 2.11. Oxidative Damage

Reactive oxygen species (ROS) constitute an important class of DNA damaging agents, and their generation can be elevated due to exposure to tobacco smoke. In addition to damaging DNA directly, ROS also cause DNA damage indirectly by reacting with other macromolecules [106]. For example, ROS can lead to lipid peroxidation of polyunsaturated fatty acids and produce malondialdehyde, which reacts with DNA to form DNA adducts, with the predominant one being 3-(2-deoxy-β-d-erythro-pentafuranosyl)pyrimido[1,2-α]purin-10(3*H*)-one deoxyguanosine (M_1_dG, Figure 2) [107]. In addition to M_1_dG, some representative oxidative damage-associated DNA adducts include 8-oxo-dG, 1,*N*^6^-etheno-2-deoxyadenosine (εdA), and 3,*N*^4^-etheno-2′-deoxycytidine (εdC). A more comprehensive summary of the formation and biological consequences of oxidative stress-induced DNA damage was covered by a recent review [106].

M_1_dG is a premutagenic lesion and induces G→T and G→A mutations, which could be an important step in the etiology of oxidative associated-diseases. M_1_dG was detected in human leukocytes [33,48,108,109,110,111] and nasal epithelium (Table 1) [112,113]. Studies suggest that smoking contributes to the levels of M_1_dG in humans. In a study of workers occupationally exposed to silica dust, the levels of M_1_dG in nasal epithelium of smokers (*n* = 58) and former smokers (*n* = 63) were 78 ± 9.8 and 81 ± 9.7 adducts per 10^8^ nucleotides, respectively, significantly higher (*p* < 0.05) compared to nonsmokers (*n* = 132) with M_1_dG levels of 57 ± 6.2 adducts per 10^8^ nucleotides [112]. In a study of occupational exposure to asbestos, higher M_1_dG levels (*p* = 0.005) were observed in leukocytes of heavy smokers (>40 packs/year, *n* = 12) compared to nonsmokers (*n* = 136), but the difference was not observed among moderate smokers (20.1–40 packs/year, *n* = 29), light smokers (0.1–20 packs/year, *n* = 20), and nonsmokers [108]. In another study investigating the effects of diet on M_1_dG levels among industrial estate workers, no difference was observed between smokers (*n* = 46) and nonsmokers (*n* = 17) among those workers. However, in the control group, the adduct levels were significantly higher (*p* < 0.05) in smokers (*n* = 64) compared to nonsmokers (n = 53) [110]. In addition to the findings that the levels of M_1_dG were elevated due to smoking, some studies also demonstrated the correlation of M_1_dG and DNA methylation in smokers [48,111].

We developed an LC-NSI-HRMS/MS method for the analysis of M_1_dG in human leukocyte DNA, and measured M_1_dG in buffy coat samples (leukocyte-containing fraction) from 25 smokers and 25 nonsmokers (Table 1). The adduct levels in smokers and nonsmokers averaged 2.2 ± 2.4 and 1.9 ± 2.0 adducts per 10^8^ nucleotides, respectively, a non-significant difference [33]. Similarly, the effect of smoking on M_1_dG formation was not significant in another two studies [109,113].

8-Oxo-dG is the most prominent and widely studied oxidative damage-related DNA adduct. It is a highly mutagenic lesion and mispairs with A during DNA replication leading to a GC→AT conversion. 8-Oxo-dG was detected in various human sample types, including semen [114,115], retina [116], leukocytes [117,118,119,120], and urine samples (Table 1) [121]. In a study investigating the effect of tobacco use on the possible etiology of childhood cancer, 8-oxo-dG was measured by an immunochemical method in semen samples from the children’s fathers. The levels of 8-oxo-dG were 66 ± 2.9, 54 ± 4.4, and 179 ± 20 ng/mL in smokers (*n* = 33), tobacco chewers (*n* = 31), and subjects who both smoked and chewed tobacco (*n* = 41), respectively, all significantly higher than nonsmokers (*n* = 33) with adduct levels of 34 ± 1.1 ng/mL [115]. The effect of smoking on 8-oxo-dG formation was either not significant or not investigated in other studies [114,116,117,118,119,120,121].

εdA and εdC are two representative DNA adducts formed by 4-hydroxy-2-nonenal, a product of lipid peroxidation. Both adducts are promutagenic and could be useful markers to assess oxidative stress-derived DNA damage. Using an LC-ESI-MS/MS method, Bin, et al measured εdA and εdC in urine to investigate the effect of occupational exposure to diesel engine exhaust on adduct formation (Table 1) [122]. The levels of εdA averaged 0.19 nmol/g creatinine in exposed workers (*n* = 108), significantly higher (*p* < 0.001) than the control group (*n* = 109) with εdA levels averaging 0.09 nmol/g creatinine. The levels of εdC showed no difference between the two groups. The contribution of smoking to the formation of εdA or εdC was not significant [122].

## 3. Newly Characterized Tobacco-Specific DNA Adducts

Since 2012, multiple DNA adducts formed by tobacco-specific carcinogens (i.e., NNK and NNN) have been characterized and quantified in in vitro models and in NNK- or NNN-treated animals [19,20,21,31,34,35,36,123,124]. In addition to DNA base adducts, which have been extensively studied, we have recently characterized and measured a panel of DNA phosphate adducts formed by NNK and NNN [20,34,35,36]. The biological significance of these newly identified DNA adducts is still largely unknown and further studies need to be conducted to better understand the formation, removal, and biological implications of these DNA adducts to facilitate their use as biomarkers in exposure evaluation and cancer risk assessment.

### 3.1. DNA Phosphate Adducts

In addition to DNA base moieties, some alkylating agents also react with the oxygen of the phosphate backbone to form DNA phosphate adducts [125]. Earlier studies have demonstrated that phosphate adducts formed by certain carcinogens persisted in vivo and had longer half-lives than their corresponding base adducts [126,127], suggesting DNA phosphate adducts may serve as better biomarkers of chronic exposure to those carcinogens. For the formation of DNA phosphate adducts by the tobacco-specific carcinogens NNK and NNN, there was only one study using a transalkylation approach to indirectly measure the adduct levels in [^3^H]NNK-treated mice [128]. However, that study only provided indirect proof of the presence of NNK-derived DNA phosphate adducts, and the chemical structures were not characterized for individual adducts.

One major challenge of direct measurement of DNA phosphate adducts is their structural complexity. After enzyme hydrolysis of DNA samples, DNA phosphate adducts are measured as phosphotriesters (PTE)—B_1_p(alkyl)B_2_. B_1_ and B_2_ are the same or different nucleosides, which can be 10 different combinations of the four nucleosides (Table 2) [125]. Due to the tetrahedral phosphate group in B_1_p(alkyl)B_2_, there can be *R*p or *S*p diastereomers present. With the same nucleosides, there can be two isomers depending on which oxygen is alkylated. With different nucleosides, because of the connection of sugar moieties, it can be either B_1_-5′-alkyl-3′-B_2_ or B_1_-3′-alkyl-5′-B_2_. Therefore, there can be 32 different isomers of B_1_p(alkyl)B_2_ (Table 2). In order to characterize and measure all of the 32 possible combinations, a specific and powerful analytical method is required. We have developed a series of LC-NSI-HRMS/MS-based methods and characterized four different types of NNK- and NNN-derived DNA phosphate adducts, including pyridyloxobutyl DNA phosphate adducts [B_1_p(POB)B_2_], pyridylhydroxybutyl DNA phosphate adducts [B_1_p(PHB)B_2_: B_1_p(PHB)_s_B2 + B_1_p(PHB)_b_B_2_], and methyl DNA phosphate adducts [B_1_pMeB_2_] [20,34,35,36,124].

#### 3.1.1. B_1_p(POB)B_2_

One metabolic pathway of NNK proceeds via α-hydroxylation of its methyl group to produce the unstable intermediate **6** (Figure 3). This intermediate spontaneously yields **11** and then the alkylating agent **14**, which reacts with DNA to form POB DNA base adducts and POB DNA phosphate adducts—B_1_p(POB)B_2_ (Figure 3) [20]. We first characterized and detected B_1_p(POB)B_2_ in calf thymus DNA (CT-DNA) treated with 4-(acetoxymethylnitrosamino)-1-(3-pyridyl)-1-butanone (NNKOAc, **2**), a regiochemically activated form of NNK. The structures of two combinations of B_1_p(POB)B_2_, Cp(POB)C and Tp(POB)T, were confirmed by comparing with the synthetic standards [20,36], and the identities of the other B_1_p(POB)B_2_ adducts were confirmed on the basis of the accurate masses of the precursor ions and the corresponding fragment ions. A total of 30 out of 32 possible B_1_p(POB)B_2_ adducts were detected in NNKOAc-treated CT-DNA. The B_1_p(POB)B_2_ adducts were also detected and quantified in rats treated with NNK acutely (0.1 mmol/kg once daily for 4 days by subcutaneous injection) and chronically (5 ppm in drinking water for 10, 30, 50, and 70 weeks). In the chronically treated rats, some B_1_p(POB)B_2_ adducts are persistent and abundant over 70 weeks, which suggested that those adducts could potentially be detected in smokers and be used as biomarkers to investigate chronic exposure to NNK [20]. The B_1_p(POB)B_2_ adducts were also detected in rats treated with NNAL [35] or NNN [124].

#### 3.1.2. B_1_p(PHB)B_2_

NNK is extensively metabolized to NNAL, which undergoes α-hydroxylation to produce diazonium ion **16**. Diazonium ion **16** reacts with DNA to form PHB base adducts and PHB DNA phosphate adducts, B_1_p(PHB)_s_B_2_, where the subscript s represents ‘straight chain’ (Figure 3). Diazonium ion **16** further rearranges to carbocation **17**, which also reacts with DNA to form another type of PHB DNA phosphate adducts—B_1_p(PHB)_b_B_2_, where the subscript b represents ‘branched chain’ (Figure 3). Since both B_1_p(PHB)_s_B_2_ and B_1_p(PHB)_b_B_2_ are derived from NNAL, they are collectively called B_1_p(PHB)B_2_ phosphate adducts [36]. For B_1_p(PHB)_s_B_2_, because of the two chiral centers, the phosphorus and carbinol carbon, there can be four and eight isomers with the same and different nucleoside combinations, respectively. Therefore, a total of 64 different possible B_1_p(PHB)_s_B_2_ adducts can be formed (Table 2). Compared to B_1_p(PHB)_s_B_2_, an additional chiral center at the methyl-bearing carbon is present in the structure of B_1_p(PHB)_b_B_2_ adducts, so there can be eight and 16 isomers with the same and different nucleoside combinations, respectively. Consequently, a total of 128 different possible B_1_p(PHB)_b_B_2_ adducts can be formed (Table 2). Therefore, a total of 192 possible B_1_p(PHB)B_2_ adducts can be formed from the α-hydroxylation of NNAL’s methyl group. Using a similar strategy as in B_1_p(POB)B_2_ characterization, a total of 107 out of 192 possible B_1_p(PHB)B_2_ adducts were detected in the NNK-treated rat lung DNA samples. Similar to B_1_p(POB)B_2_ adducts, certain B_1_p(PHB)B_2_ adducts demonstrated persistence for over 70 weeks, suggesting that they could be potential biomarkers of chronic exposure to NNK and NNAL [36]. Both B_1_p(POB)B_2_ and B_1_p(PHB)B_2_ adducts were also detected in NNAL-treated rats [35].

#### 3.1.3. B_1_pMeB_2_

In addition to the methyl group, α-hydroxylation also occurs on the α-methylene carbon of NNK to produce methane diazohydroxide **15**, which reacts with DNA to form methyl DNA base and phosphate adducts (B_1_pMeB_2_) [34]. Similar to B_1_p(POB)B_2_ adducts, there are 32 possible isomers of B_1_pMeB_2_ phosphate adducts (Table 2). We characterized and detected B_1_pMeB_2_ adducts in lung DNA of rats treated with NNK in their drinking water (5 ppm) for 10, 30, 50, and 70 weeks, and a total of 23 out of 32 possible isomers were detected. Thus far, we have identified 30 B_1_p(POB)B_2_ adducts and 107 B_1_p(PHB)B_2_ adducts, resulting in a total of 160 different structurally unique DNA phosphate adducts in NNK-treated rats. This is by far the most structurally diverse panel of DNA adducts identified from any carcinogen. Consistent with the observations of B_1_p(POB)B_2_ and B_1_p(PHB)B_2_ adducts, certain B_1_pMeB_2_ phosphate adducts showed persistence in NNK-treated rats. B_1_pMeB_2_ adducts were also detected in NNAL-treated rats in our study [34]. The relative levels of B_1_p(POB)B_2_, B_1_p(PHB)B_2_ and B_1_pMeB_2_ phosphate adducts, and their corresponding base adducts in lung DNA of rats treated with NNK or NNAL were summarized in that study.

### 3.2. DNA Base Adducts

In addition to DNA phosphate adducts, multiple DNA base adducts that are associated with NNK and NNN were either newly characterized in vitro or detected for the first time in vivo. These DNA adducts include 2-(2-(3-pyridyl)-*N*-pyrrolidinyl)-2′-deoxyinosine (py-py-dI) [31], 3-POB-dC, *N*^4^-POB-dC [21], *O*^4^-POB-dT [123], *N*^6^-POB-dA, and *N*^1^-POB-dI [19].

#### 3.2.1. Py-py-dI

Metabolic activation of NNN occurs via one of two pathways: 2′-hydroxylation or 5′-hydroxylation (Figure 3), and 5′-hydroxylation is likely to be the major metabolic pathway in humans. The major adduct formed in vitro via 5′-hydroxylation was py-py-dI, which forms upon the reaction of the *N*^2^ position of dG with diazonium ion **13** followed by NaBH_3_CN reduction [129]. Using an LC-ESI-MS/MS method, we have recently detected py-py-dI in rats treated with NNN in the drinking water (7–500 ppm). Py-py-dI was the major DNA adduct resulting from 5′-hydroxylation, with the highest levels being detected in the lung and nasal cavity of NNN-treated rats. Our study also showed that py-py-dI was more abundant than the 2′-hydroxylation of NNN-derived POB DNA base adducts in the in vitro studies in which human liver S9 fraction or human hepatocytes were incubated with NNN (2–500 μM) [31]. The results of this study identified py-py-dI as the major NNN-derived DNA adduct in the human enzyme systems examined. Thus, py-py-dI could be a useful biomarker to investigate the metabolic activation of NNN in humans.

#### 3.2.2. 3-POB-dC and N^4^-POB-dC

The intermediate **14** from NNK also reacts with dC in DNA and forms *O*^2^-POB-C, which has been detected in rats treated with NNK or NNN. In addition to the *O*^2^-position, the 3-position and exocyclic *N*^4^-amino group of dC are also potentially reactive sites for alkylation, possibly forming 3-POB-dC and *N*^4^-POB-dC, respectively. We have recently synthesized the chemical standards of 3-POB-dC and *N*^4^-POB-dC, and confirmed the presence of both adducts in NNKOAc-treated CT-DNA. In agreement with our previous studies, *O*^2^-POB-C was the most abundant dC adduct, while considerably lower amounts of 3-POB-dC and *N*^4^-POB-dC were present in NNKOAc-treated CT-DNA [21]. The results of this study provide a more complete picture of dC adduct formation in the reaction of NNKOAc with DNA.

#### 3.2.3. O^4^-POB-dT

The major dT adduct formed by NNK and NNN is *O*^2^-POB-dT. Using an LC-NSI-MS/MS method, Leng, et al detected and characterized for the first time a new dT adduct—*O*^4^-POB-dT, in NNKOAc-treated CT-DNA [123]. The identity of *O*^4^-POB-dT was confirmed by the chemical standard synthesized in the study. *O*^4^-POB-dT was also detected in human skin fibroblasts and Chinese hamster ovary cells treated with NNKOAc. *O*^2^-POB-dT was detected in all the systems with higher levels than *O*^4^-POB-dT. Results from this study indicated that both *O*^4^-POB-dT and *O*^2^-POB-dT were subjected to repair by the nucleotide excision repair pathway [123].

#### 3.2.4. dA Adducts

All the previous studies have identified and measured a panel of POB DNA base adducts of dG, dC, and dT, but not dA. To complete the panel of POB DNA base adducts, we determined the possible formation of POB-dA adducts in the in vitro NNKOAc-treated CT-DNA and in vivo NNK-treated rats [19]. We hypothesized that the initial alkylation of dA would occur at the *N*^1^ position (Figure 3), which results in an unstable cationic intermediate adduct. This unstable adduct can either deprotonate and undergo spontaneous Dimroth rearrangement to give *N*^6^-POB-dA, or deaminate via an addition−elimination mechanism to give *N*^1^-POB-dI. In support of our hypothesis, both *N*^6^-POB-dA and *N*^1^-POB-dI were detected in NNKOAc-treated CT-DNA using our newly developed LC-ESI-MS/MS method. In contrast, only *N*^6^-POB-dA but not *N*^1^-POB-dI was detected in lung and liver DNA from rats treated with NNK for 50 weeks (5 ppm in the drinking water), possibly due to the rapid repair of *N*^1^-POB-dI. Similarly, we also detected N6-PHB-dA in the NNAL-treated rats [19].

## 4. Perspectives

### 4.1. Current Limitations

Among all the adducts described in this review, some have been found to be at the same or overlapping levels in smokers and nonsmokers, while others had significantly higher levels in smokers compared to nonsmokers. One should keep in mind when investigating the effects of smoking on DNA adduct formation, the association may be compromised by a number of confounding variables, such as current smoking status at the time of sample collection, exposures to secondhand smoke, air pollution, occupational exposure, dietary habits, and disease status. For example, self-reported smoking status collected through questionnaires may not be accurate. In addition, in studies which used surgically obtained tissues from cancer patients, smoking status may not be up-to-date if subjects stopped smoking for a few weeks or more before surgery. This would have an unknown effect on DNA adduct levels since their persistence in those human tissues is generally unknown. To overcome this limitation, urine samples can be collected from the same subjects for the measurement of tobacco biomarkers (e.g., NNAL and cotinine) to confirm their current smoking status.

Another potential confounding variable is the presence in the environment of the same chemicals that form DNA adducts. For example, PAH are tobacco smoke carcinogens and DNA adduct formation by PAH is often used to investigate smoking-related cancers. However, PAH are also present in the environment where combustion occurs and in the diet due to high temperature cooking. Therefore, factors such as air pollution and diet need to be taken into account when interpreting the association between PAH-derived DNA adduct formation and tobacco smoke exposure. The only carcinogens that are specific to tobacco smoke are the tobacco-specific nitrosamines such as NNK and NNN [7,15]. DNA adducts formed by NNK and NNN therefore have the potential to be ideal biomarkers to investigate tobacco-related cancer risk.

### 4.2. Analytical Methodologies

The chemical analysis of DNA adducts has been extensively reviewed [107,130,131]. To utilize DNA adducts as biomarkers of tobacco smoke exposure and cancer risk assessment, reliable analytical methods have to be developed and fully validated for linearity, accuracy, precision, specificity, and ruggedness. High-throughput methods are preferred and will allow the analysis of DNA adducts to be performed in studies with large sample sizes, which are typical in epidemiology. ^32^P-postlabeling and immunochemical approaches were extensively used for DNA adduct analysis in the past and are still quite prevalent nowadays. However, the uncertainty in labeling efficiency and the lack of physiochemical structural confirmation of the ^32^P-postlabeling technique are major disadvantages potentially undermining the adequacy of data produced by this method. A critical drawback of the immunochemical approach is that the specificity of the antibodies for some DNA adducts is uncertain, as they may cross-react with other DNA lesions or endogenous components, resulting in errors in characterization and quantitation. The disadvantages of ^32^P-postlabeling and immunochemical approaches are evident when the results are compared to those obtained by mass spectrometry-based methods. For example, the levels of BPDE-*N*^2^-dG and 4-ABP-C8-dG, as discussed in this review, and the levels of DNA adducts formed by 2-amino-1-methyl-6-phenylimidazo[4,5-*b*]pyridine [65], were all generally 10 to 100-fold higher in the same type of tissues when results of ^32^P-postlabeling or immunochemical methods were compared to those obtained by using mass spectrometry-based methods. This difference suggests that one should be cautious when interpreting data obtained from ^32^P-postlabeling or immunochemical methods. We recommend that DNA adduct identity and quantity be confirmed by well-validated mass spectrometry-based methods.

During the past few decades, mass spectrometry coupled with the stable isotope dilution methods has evolved to become the gold standard for identification, characterization, and quantitation of DNA adducts. Further development and validation of mass spectrometry-based methods for high-throughput, sensitive and accurate quantitation of DNA adducts in humans are required in future studies. The LC-NSI-HRMS/MS-based technique can achieve detection limits in the low amol (10^−18^ mol), or even high zmol (10^−21^ mol) range, allowing the highest levels of specificity and sensitivity. To achieve such detection limits in human biological samples, the samples should be thoroughly purified to avoid co-eluting interferences and ion suppression during the mass spectrometry analysis. In addition to the analysis of targeted DNA adducts, mass spectrometry-based DNA adductomics can be used to comprehensively screen for DNA modifications, including both known and unknown/unidentified DNA adducts, and expedite the discovery of novel tobacco smoke-related DNA adducts [132].

### 4.3. Artifactual Formation

Particularly for DNA adducts formed endogenously, in addition to the efforts on improving the detection sensitivity and sample purification process, precautions should be taken to eliminate artifactual formation during all the steps of sample analysis, including sample collection and storage, DNA isolation and enzymatic hydrolysis, sample purification and enrichment, and mass spectrometry analysis. For instance, artifactual formation is one likely contributor to the relatively high levels of γ-OH-Acr-dG and Cro-dG measured in one study [53]. Another example is 8-oxo-dG. When the DNA is extracted from the cell, the in vivo protecting enzymes and antioxidants are no longer present to neutralize oxygen radicals and repair DNA damage, and 8-oxo-dG can thus be formed by the oxidation of free dG as well as dG in DNA [133].

We suggest the following strategy to systematically evaluate and reduce the possible artifactual formation of endogenous DNA adducts: 1) Sample collection. The samples should be handled by well-trained personnel to reduce the period between collection and storage. The samples should be placed on ice if they cannot be processed immediately; 2) Sample storage. The storage conditions should be evaluated to determine that no artifactual formation or degradation of DNA adducts occurs. A comparison of DNA adduct levels can be performed between the DNA isolated immediately after sample collection and the DNA isolated from the same sample after a certain period of storage; 3) DNA isolation. Commercially available calf thymus DNA (CT-DNA) contains most of the endogenous DNA adducts at certain levels, and can be used to investigate possible artifactual formation. To perform such an experiment, CT-DNA is dissolved in solution and isolated using the proposed protocol [33,116]. The adduct levels in the isolated CT-DNA are then compared to the levels in the same CT-DNA without isolation to determine whether the adduct levels are impacted by the DNA isolation process. This approach can also be used to investigate whether certain chemicals (e.g., antioxidants) can be used to lower or prevent artifactual formation; 4) DNA enzymatic hydrolysis and sample purification. Stable isotope-labeled nucleotides can be added to samples prior to starting DNA hydrolysis, and the possible artifactual formation can be monitored by checking whether correspondingly labeled DNA adducts are present [116]; 5) Mass spectrometry analysis. Depending on the sample purification approach, the free nucleotides may still be in the final samples and form corresponding adducts in the ion source of the mass spectrometer (e.g., dG forms 8-oxo-dG in the ESI ion source) [116]. If the free nucleotide happens to co-elute with the adduct, additional adducts may be formed from the free nucleotide, resulting in the overestimation of the adduct levels. To avoid this, the free nucleotide should be separated from the adduct, either at the sample purification step or on the LC column during the mass spectrometry analysis.

### 4.4. Other Considerations

Stability of DNA adducts over time. The adduct levels are supposed to reflect the balance between DNA adduct formation and repair. If this is the case, it would be expected that the DNA adduct level would remain relatively constant over time in a similar exposure environment. In the case of tobacco smoke-related DNA adducts, the level of an ideal DNA adduct as a biomarker should stay constant if the smoker maintains his/her habit. A longitudinal study can be performed where the same biological samples are collected at different time intervals (days, weeks, or months apart) and the level of this DNA adduct is measured to determine its stability over time.

Surrogate tissues. To conduct a longitudinal study, surgically obtaining human tissues such as lung becomes highly impractical. Instead, surrogate tissues such as bronchoalveolar lavage (BAL), peripheral white blood cells or oral cells can be used. For example, studies have demonstrated a correlation between changes in the oral cavity and lung in smokers [26,134], so oral cells would potentially be a source of readily obtained biological samples.

Computational approach. In addition to experimental techniques, computational approaches have been used to study conformational preference of the adducts in DNA, their potential to cause mismatch and mutations, and their interactions with DNA polymerases and repair enzymes [135,136,137]. For example, a multiscale computational approach demonstrated that both *O*^6^-POB-G and *O*^6^-PHB-G pair with C and T, with the latter being more mutagenic because of the difference in the bulky moiety hydrogen-bonding pattern [135]. Such studies provide important insights into the biological significance of smoking-related DNA adducts, which is critical for understanding their role in tobacco-induced cancers.

## Figures and Tables

**Table 1:** Detection of tobacco smoke-related DNA adducts in human samples.

DNA Adduct	Source	Tissue	Method of Detection	Reference
HPB-releasing DNA adducts	NNK, NNN	Buccal cells	LC-NSI-HRMS/MS, LC-MS/MS	[28,29]
Bulky adducts	PAH, other aromatic and nonpolar chemicals (?)	Leukocytes, lung	^32^P-postlabelling	[39,40,41,42,43,44,45,46,47,48,49,50,51,52]
BPDE-*N^2^*-dG	Benzo[*a*]pyrene	Buccal cells, sputum, lung, leukocytes, lymphocytes, tonsil, oropharynx, gingiva, prostate	Immunochemical, LC-NSI-HRMS/MS	[32,53,54,55,56,57,58]
4-ABP-C8-dG	4-aminobiphenyl (4-ABP)	Bladder, breast	LC-NSI-HRMS/MS, ^32^P-postlabelling, LC-ESI-MS/MS^3^	[51,67,68]
4-ABP-C8-dA	4-ABP	Bladder	^32^P-postlabelling	[68]
4-ABP-*N*^2^-dG	4-ABP	Bladder	^32^P-postlabelling	[68]
*O*^6^-methyldeoxyguanosine (*O*^6^-mdG)	NNK, NDMA, other methylating agents	Lung	Immunochemical	[53]
7-Methylguanine (7-mG)	NNK, NDMA, other methylating agents	Lung, urine,	Capillary LC-HRMS/MS, Immunochemical	[74,75,78]
3-Methyladenine (3-mA)	NNK, other methylating agents	Urine	Capillary LC-HRMS/MS, LC-ESI-MS/MS	[75,76,77,78]
1-Methyladenine (1-mA)	NNK, other methylating agents	Urine	Capillary LC-HRMS/MS	[75]
Methyl DNA phosphate adducts (B_1_pMeB_2_)	NNK, other methylating agents	Lung	LC-NSI-HRMS/MS	[79]
3-Ethyladenine (3-etA)	Unknown ethylating agents	Urine, saliva	LC-ESI-MS/MS, LC-NSI-MS/MS	[76,77,87]
7-Ethylguanine (7-etG)	Unknown ethylating agents	Urine, leukocytes, saliva	LC-ESI-MS/MS, LC-NSI-MS/MS	[76,84,87,88]
*O*^2^-ethylthymidine (*O*^2^-etT)	Unknown ethylating agents	Leukocytes, saliva	LC-NSI-MS/MS	[83,86]
*O*^4^-ethylthymidine (*O*^4^-etT)	Unknown ethylating agents	Leukocytes, saliva	LC-NSI-MS/MS	[83,86]
*N*^3^-ethylthymidine (*N*^3^-etT)	Unknown ethylating agents	Leukocytes, saliva	LC-NSI-MS/MS	[83,84,86,88]
7-(2, 3, 4-trihydroxybut-1-yl) guanine (7-THBG)	1,3-Butadiene	Leukocytes	Capillary LC-HRMS/MS	[90]
1,*N^2^*-propanodeoxyguanosine (Acr-dG)	Acrolein	Buccal cells, sputum, lung, saliva, bladder, liver	Immunochemical, ^32^P-postlabelling, LC-MS/MS, LC-NSI-HRMS/MS	[53,68,94,95,96]
*N^6^*-hydroxymethyldeoxyadenosine (HOMe-dA)	Formaldehyde	Saliva	LC-ESI-MS/MS	[95]
*N^2^*-hydroxymethyldeoxyguanosine (HOMe-dG)	Formaldehyde	Saliva	LC-ESI-MS/MS	[95]
*N^2^*-ethylidene-deoxyguanosine (Ethylidene-dG)	Acetaldehyde	Saliva, leukocytes, oral cells	LC-ESI-MS/MS	[95,101,102,103]
3-(2′-Deoxyribos-1′-yl)-5,6,7,8-tetrahydro-8-hydroxy-6-methylpyrimido[1,2-*a*]purine-10(3*H*)one (Cro-dG)	Crotonaldehyde, acetaldehyde	Buccal cells, sputum, lung, urine	Immunochemical, LC-ESI-MS/MS	[53,100]
7-(2-carbamoyl-2-hydroxyethyl) guanine (7-GAG)	Acrylamide	Urine	LC-ESI-MS/MS	[104,105]
3-(2-deoxy-β-d-erythro-pentafuranosyl)pyrimido[1,2-α]purin-10(3H)-one deoxyguanosine (M_1_dG)	Malondialdehyde	Leukocytes, nasal epithelium	^32^P-postlabelling, LC-NSI-HRMS/MS	[33,48,108,109,110,111,112,113]
8-Oxo-dG	ROS	Semen, retina, leukocytes, urine	Immunochemical, LC-NSI-MS/MS, electrochemical	[114,115,116,117,118,119,120,121]
1,*N^6^*-etheno-2′-deoxyadenosine (εdA)	4-hydroxy-2-nonenal	Urine	LC-ESI-MS/MS	[122]
3,*N^4^*-etheno-2′-deoxycytidine (εdC)	4-hydroxy-2-nonenal	Urine	LC-ESI-MS/MS	[122]

**Table 2:** Numbers of possible isomers of NNK-derived DNA phosphate adducts.

B_1_-B_2_	Number of Possible Isomers				Total
	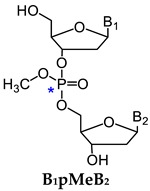	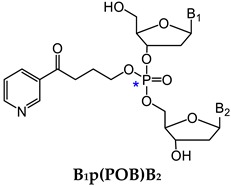	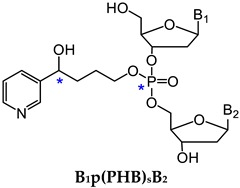	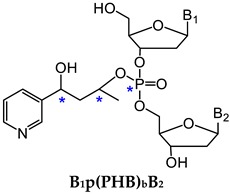	
A-A	2	2	4	8	16
C-C	2	2	4	8	16
G-G	2	2	4	8	16
T-T	2	2	4	8	16
A-C	4	4	8	16	32
A-G	4	4	8	16	32
A-T	4	4	8	16	32
C-G	4	4	8	16	32
C-T	4	4	8	16	32
G-T	4	4	8	16	32
Total	32	32	64	128	256

* Chiral center.
